# Comparative analysis of the intestinal bacterial communities in mud crab *Scylla serrata* in South India

**DOI:** 10.1002/mbo3.1179

**Published:** 2021-05-01

**Authors:** Elina Apine, Praveen Rai, Madhu K. Mani, Vikram Subramanian, Indrani Karunasagar, Anna Godhe, Lucy M. Turner

**Affiliations:** ^1^ Marine Biology and Ecology Research Centre University of Plymouth Plymouth UK; ^2^ Nitte University Centre for Science Education and Research (NUCSER) Nitte (Deemed to be University) Mangaluru India; ^3^ Biokart India Pvt Ltd Bengaluru India; ^4^ Department of Marine Sciences University of Gothenburg Gothenburg Sweden

**Keywords:** 16S rRNA, aquaculture, bacterial diversity, gut microbiome, mud crab, nanopore sequencing

## Abstract

Little is known about the functions of the crustacean gut microbiome, but environmental parameters and habitat are known to affect the composition of the intestinal microbiome, which may in turn affect the physiological status of the host. The mud crab *Scylla serrata* is an economically important species, and is wild‐caught, and farmed across the Indo‐Pacific region. In this study, we compared the composition of the gut microbiome (in terms of gut microbial species richness and abundance) of *S. serrata* collected from wild sites, and farms, from the east and west coast of India, and also tested the effects of the environment on the composition. The water temperature had a statistically significant effect on gut microbiome composition, with microbial biodiversity decreasing with increasing water temperature. This could have negative effects on both wild and farmed mud crabs under future climate change conditions, although further research into the effects of temperature on gut microbiomes is required. By comparison, salinity, crab mass and carapace width, geographical location as well as whether they were farmed or wild‐caught crabs did not have a significant impact on gut microbiome composition. The results indicate that farming does not significantly alter the composition of the gut microbiome when compared to wild‐caught crabs.

## Graphical abstract

The effects of habitat (wild vs farmed), location, and environment on the composition of the gut microbiome of the mud crab *Scylla serrata* in India were tested. Location and habitat are not associated with differences in intestinal microbial composition. However, the water temperature had a significant effect and microbial diversity decreases with increasing temperature. This could have negative effects on both wild and farmed mud crabs under future climate change conditions.

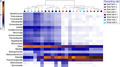

## 1 INTRODUCTION

It is now well established that the gut microbiome of humans and other vertebrates is involved in various physiological processes such as development, nutrition, and the immune response, including the production of vitamins and exogenous enzymes (e.g. Belkaid & Hand, 2014; Brestoff & Artis, 2013; Rowland et al., 2018), all of which play an important role in maintaining the internal environment of the host. Whilst it has been hypothesized that the crustacean gut microbiome positively contributes to crustacean physiological and metabolic status (Cornejo‐Granados et al., 2018), and any disturbance in the delicate balance of the gut microbial composition can affect their susceptibility to pathogens (Shi et al., 2019), relatively little is known about the structure and function of the intestinal microbiota in this group. The composition of the crustacean gut microbiome depends on several internal and external factors such as the developmental stage of the host (e.g. Rungrassamee et al., 2013), host anatomy (e.g. Apprill, 2017) environmental conditions that are either seasonal or sudden and extreme events such as prevalent rainfalls, increased temperature, as well as their habitat, availability of feed (e.g. Sullam et al., 2012; Xia et al., 2014) and stress related to, for instance, territorialism (Moloney et al., 2014).

Crabs from the genus *Scylla* are currently the only farmed commercial crab species in India and the mud crab *Scylla serrata* is a particularly economically important species due to its large size and high meat content (Le Vay, 2001). Crab farming is a growing sector, especially in the state of Andhra Pradesh on the east coast that is considered the “cradle of Indian aquaculture” (Belton et al., 2017). On the other hand, local communities along the state of Karnataka on the west coast are involved in sporadic marine and inland fishing, rather than the farming of crabs on a large scale (Government of Karnataka, 2016). Studies on fish have shown that hatchery‐reared and/or captive fish have microbiomes that differ from their wild counterparts with reduced biodiversity or significantly different composition that potentially can lead to disadvantages to the host, such as altered metabolic pathways, and reduced immunity (e.g. Lavoie et al., 2018; Ramirez & Romero, 2017).

In this study, we characterized the gut microbiome of the mud crab *S. serrata*, and compared the microbial composition in animals from wild sites and crab farms, from the east and west coast of India. To quantify any differences in the microbiome of crabs, we used long read 16S rRNA nanopore sequencing. Further, we aimed to examine the role of geographical location, habitat (estuaries or aquaculture pond), and environmental conditions (salinity and temperature) on their impact on gut microbial diversity and quantity and how it relates to the physiological status of the animal.

## 2 MATERIALS AND METHODS

### 2.1 Sample collection

**FIGURE 1:**
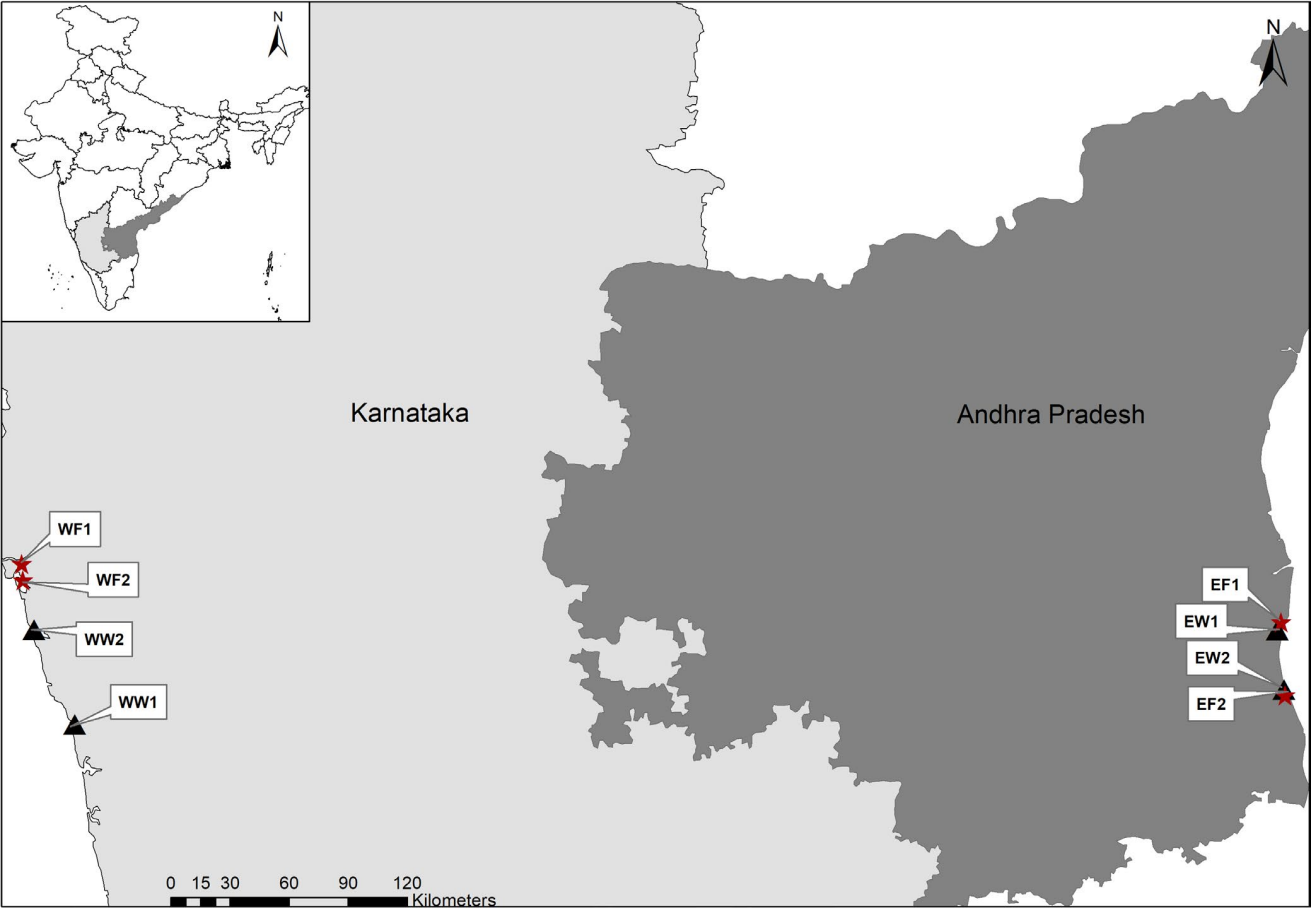
Sampling sites: triangle—wild sites, star—farms. WF—west coast farm, WW—west coast wild site, EF—east coast farm, EW—east coast wild site. Three crabs (C1‐C3) were collected from each sampling site.

Twenty four male *S. serrata* crabs (with no signs of disease) were collected from the west and east coasts of South India (Figure 1). This included animals from the wild catch and also from crab farms. Crabs (n = 3, C1‐3) from each sampling coast were collected from two sites (estuaries) representing wild samples (WW1‐2, west coast, and EW1‐2, east coast) and two culture farms (WF 1–2, west coast, and EF1‐2, east coast). Water temperature and salinity were recorded at each site (Table 1). Animals in both farms on the west coast from where samples were collected were fed with fresh bycatch, mainly sardines. Crabs on the east coast were fed with fresh tilapia in the farm EF1 and dried sardines in the farm EF2. Apart from the site EF2 where animals were fed a mix of probiotics, yeast, and jaggery (unrefined cane sugar) once a month, no additives were given at the other farms. Crabs in the sites EF2, WF1, and WF2 were kept in earthen ponds, while site EF1 was connected to the estuary. The crabs were transported to the laboratory as soon as possible and subjected to cryoanesthesia. After the measurement of weight and carapace width, the animals were thoroughly washed with sterile water and disinfected with 75% ethanol for 2–3 minutes. The animals were dissected using sterile lancets and the gut (midgut and hindgut) was separated using sterile forceps and immediately placed in sterile 1.5 mL microcentrifuge tubes. All dissecting tools were alcohol flame sterilized between dissecting each sample. Samples were stored at −80°C until further analysis.

**TABLE 1:** Characteristics of sampling sites and crabs.

Sample ID	Coast	Site type	Latitude	Longitude	Temperature (^o^C)	Salinity (ppt)	Crab mass (g)	Carapace width (mm)
WW1	West	Wild	13^o^50’53.52”N	74^o^37’52.089” E	30	27	450.88 ± 98.55	140.00 ± 14.79
WW2	West	Wild	14^o^16’47.496” N	74^o^26’37.679” E	29	33	699.56 ± 215.63	160 ± 17.32
WF1	West	Farm	14^o^34’26.364” N	74^o^22’28.938” E	28	35	148.93 ± 30.54	91.33 ± 4.93
WF2	West	Farm	14^o^30’19.296” N	74^o^23’38.151” E	27	10	815.26 ± 33.15	158.00 ± 2.00
EW1	East	Wild	14^o^16’43.86” N	80^o^7’19.436” E	31	21	200.00 ± 164.62	109.00 ± 29.51
EW2	East	Wild	14^o^0’24.948” N	80^o^9’10.411” E	30	33	103.33 ± 40.41	87.33 ± 10.11
EF1	East	Farm	14^o^18’48.168” N	80^o^8’20.893” E	27	27	366.66 ± 81.44	130.00 ± 6.55
EF2	East	Farm	13^o^58’46.272” N	80^o^9’27.586” E	35	36	190.00 ± 52.91	101.33 ± 4.16

### 2.2 DNA extraction, PCR amplification and sequencing of 16S rRNA amplicon

Total DNA from gut samples was extracted using the QIAamp DNA Stool Mini Kit (QIAGEN, Germany) and DNeasy PowerSoil Kit (QIAGEN, Germany) following the manufacturer's instructions. Intestines were firstly lysed in InhibitEX Buffer and then purified on QIAamp spin columns. Purification includes digesting proteins with Proteinase K, binding DNA to the QIAamp silica membrane, washing away impurities and eluting pure DNA from the spin column with water. The quality and quantity of extracted DNA were determined in a NanoPhotometer N60 (Implen, Germany). Samples were stored at −20°C until amplification.

The 16S rRNA gene was then amplified using forward primer 16F‐ 5’ AGAGTTTGATCMTGGCTCAG 3’ and the reverse primer 16R‐ 5’ TACGGYTACCTTGTTACGACTT 3’. The PCR mixture contained high‐fidelity DNA polymerase, 0.5 mM dNTPs, 3.2 mM MgCl_2_ and PCR enzyme buffer, 40 ng of extracted DNA and 10 pM of each primer. The reaction conditions included an initial denaturation at 95°C for 3 minutes followed by 25 cycles each of denaturation at 95°C for 15 seconds, annealing at 60°C for 15 seconds and elongation at 72°C for 2 minutes followed by a final extension at 72°C for 10 minutes. The PCR products were purified using the QIAGEN Gel Purification Kit (QIAGEN, Germany). The amplified products were outsourced for the library preparation and Oxford Nanopore Technology (ONT) 1‐D sequencing using GridION device to the Biokart India Pvt. Ltd., Bangalore, India according to the manufacturer's instruction. Briefly, amplicons were purified using the QIAGEN Gel Purification Kit (QIAGEN, Germany). DNA concentration was estimated by using a Qubit dsDNA HS assay kit and Qubit 4.0 Fluorometer (Thermo Fisher Scientific, USA). Purified PCR products from each sample were end‐repaired and dA tailing using NEBNext Ultra II End Repair/dA‐Tailing Module (New England Biolabs, USA) was performed according to the protocol described by the manufacturer. The dA tailed PCR products were ligated with barcode adaptors using the Oxford Nanopore Native Barcode kit (EXP‐PBC096) and the Oxford Nanopore 1D Ligation Sequencing kit (SQK‐LSK109). The DNA library was loaded into a flow cell for 24–48 h run on the GridION portable sequencer for sequencing (Oxford Nanopore Technologies, UK).

### 2.3 Data analysis

After base‐calling raw FAST5 files, trimming and alignment of the reads along with the operational taxonomic unit (OTU) picking was performed using GAIA 2.0 workflow (Paytuví et al., 2019). The length of the sequences varied mainly between 100 and 1600 base pairs. Sample WF2C1 was excluded from further analyses as it was a statistically significant outlier due to low quality reads according to Grubb's test (*p* < 0.05). Alpha diversity and beta diversity at the genus level were calculated in PAST (Hammer et al., 2001). METAGENassist (Arndt et al., 2012) was used to map OTUs to phenotype. Statistical analyses and plotting were carried out in PRIMER‐E (Clarke and Gorley, 2006) and the R Studio using Bray‐Curtis similarity of square root transformed data. The genera abundant less than 1% were combined in the group designated as “Other”. Values of *p* < 0.05 were considered significant (95% confidence interval). SIMPER test was used to calculate (dis)similarity between groups using the average of Bray–Curtis dissimilarity. An unconstrained hierarchical divisive clustering routine UNCTREE was used to cluster samples based on alpha diversity. As for the multivariate analysis, we chose distance‐based linear model (DistLM) in PRIMER‐E and permutational multivariate analysis of variance (PERMANOVA) using community ecology package “vegan” in the R Studio (Oksanen et al., 2017) to evaluate the significance of environmental parameters, crab mass and carapace width, geographical location and type. Chi‐square test was used to assess associations between alpha biodiversity indices and variable factors.

## 3 RESULTS

### 3.1 The composition of the gut microbiome

**FIGURE 2:**
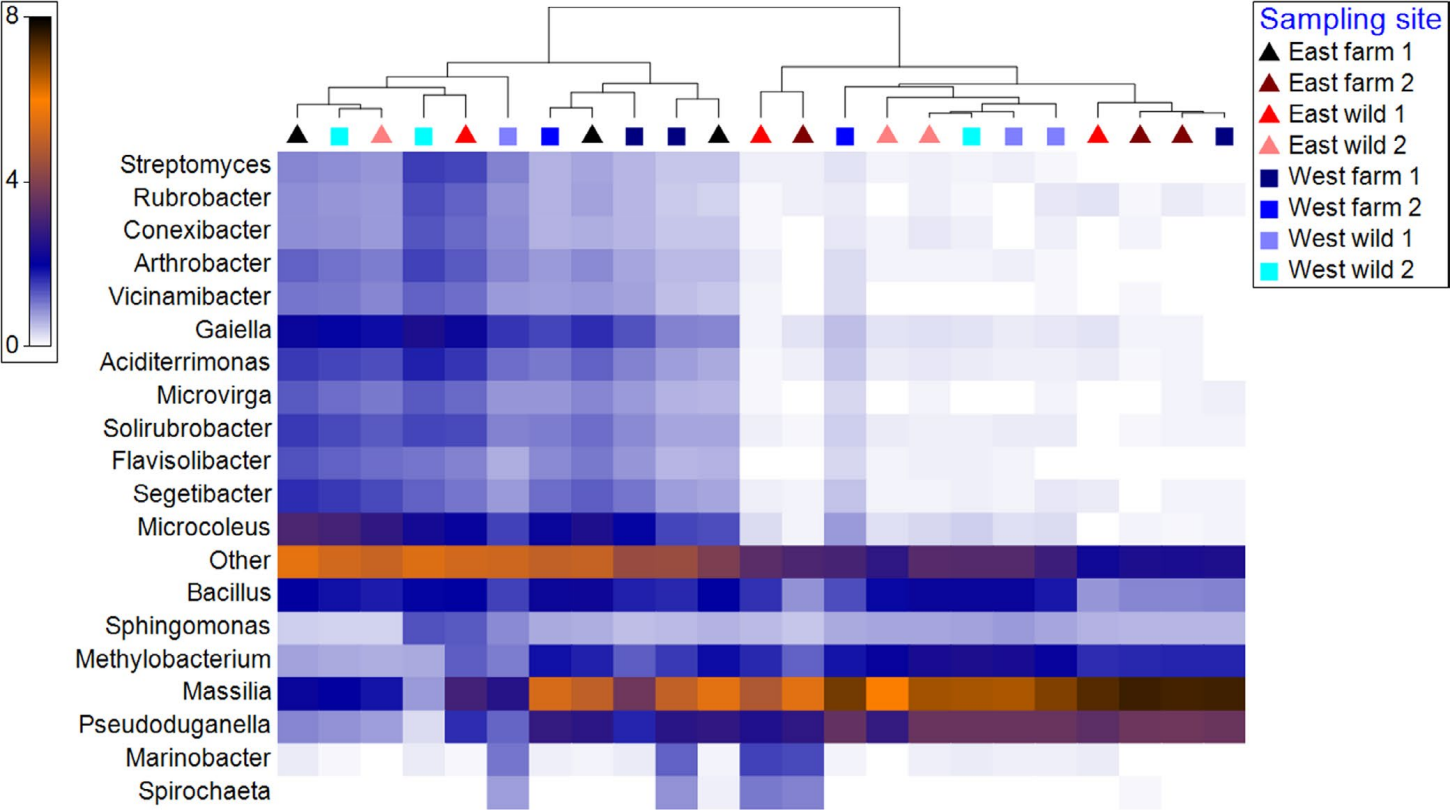
Shade plots of relative abundance of operational taxonomic units OTUs (%) assigned to 20 most abundant genera of individual crab gut microbiomes from 8 different sampling sites. Triangles represent east coast samples and squares represent west coast samples. The samples are clustered with unconstrained hierarchical divisive clustering routine UNCTREE. The relative abundance is square root transformed. The taxa present less than 1% are combined under “Other”.

The 16S rRNA amplicon sequencing on Nanopore GridION generated a total of 530,355 OTUs, from which 32% could not be assigned to any taxonomic unit. Acquired OTUs were assigned to 19 phyla, 45 classes, 88 orders, 160 families, 317 genera, and 430 species. The OTUs were assigned to five main phyla: Proteobacteria (51.8% ±9.7%), Actinobacteria (10.9% ±8.3%), Cyanobacteria, (7.3% ±4.2%) Firmicutes (4.6% ±1.1%) and Bacteriodetes (3.2% ±0.8%); five classes: *Betaproteobacteria* (43% ±12%), *Alphaproteobacteria* (5.7% ± 1.6%), *Actinobacteria* (5.1% ±3.9%), *Bacilli* (4.1% ±1.4%), and *Rubrobacteria* (3.3% ±2.0%); five major genera: *Massilia* (25% ±11.5%), *Pseudoduganella* (8.1% ±3.5%), *Microcoleus* (4.3% ±2.3%), *Bacillus* (3.1% ±1.0%), and *Gaiella* (2.9% ±1.4%) (Figure 2). At the species level, OTUs were assigned to five main species: *Massilia albidiflava* (25.2%±7.3%), *Massilia* sp. *NCCP 1146* (2.6% ±0.4%), *Microcoleus* sp. HTT‐U‐KK5 (2.6% ±1.6%), *Pseudoduganella violaceinigra* (9.3% ±2.1%), and *Aciditerrimonas ferrireducens* (1.4%± 0.9%).

**FIGURE 3:**
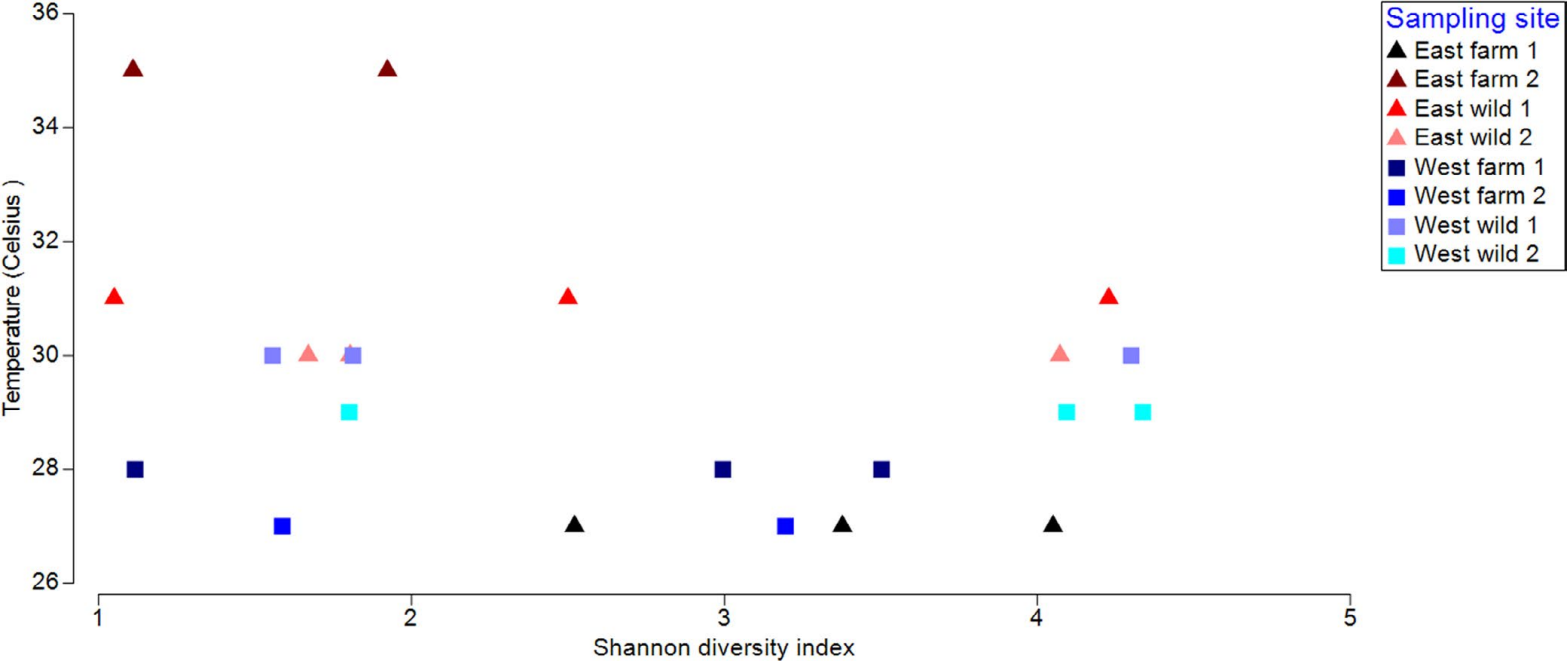
Shannon's diversity index (H’) at the genus level of individual crab gut microbiomes from 8 different sampling sites plotted against the temperature of their sampling sites. Triangles represent east coast samples and squares represent west coast samples. The samples EF2C1 and EF2C2 have similar Shannon diversity index, thus have overlapped and appear as one triangle. A higher number indicates higher biodiversity based on the OTU abundance and richness. The results of the distance‐based linear model showed that temperature had a statistically significant effect on the OTU abundance (%) at the genus level (*p* = 0.018).

Geographical location or habitat (wild or pond cultivated) did not have a significant impact on gut microbial biodiversity. On the other hand, a distance‐based linear model (DistLM) showed that temperature had a statistically significant effect on the OTU abundance (%) at the genus level (*p* = 0.018). There was a trend of decreased OTU richness with increasing temperature (Figure 3). This was also confirmed by PERMANOVA (*p* = 0.032). However, salinity, crab mass, and carapace width were not statistically significant (*p* > 0.05). Calculated alpha diversity analysis showed that the number of bacterial genera found in mud crab guts varied from 92 (EF2C1) to 289 (WW1C3). While the temperature was the only statistically significant factor that affected Shannon's diversity index (H), the number of taxa alone was also significantly affected by the coast (*p* = 0.0117) and the interaction between crab body mass and carapace width (*p* = 0.0231).

**FIGURE 4:**
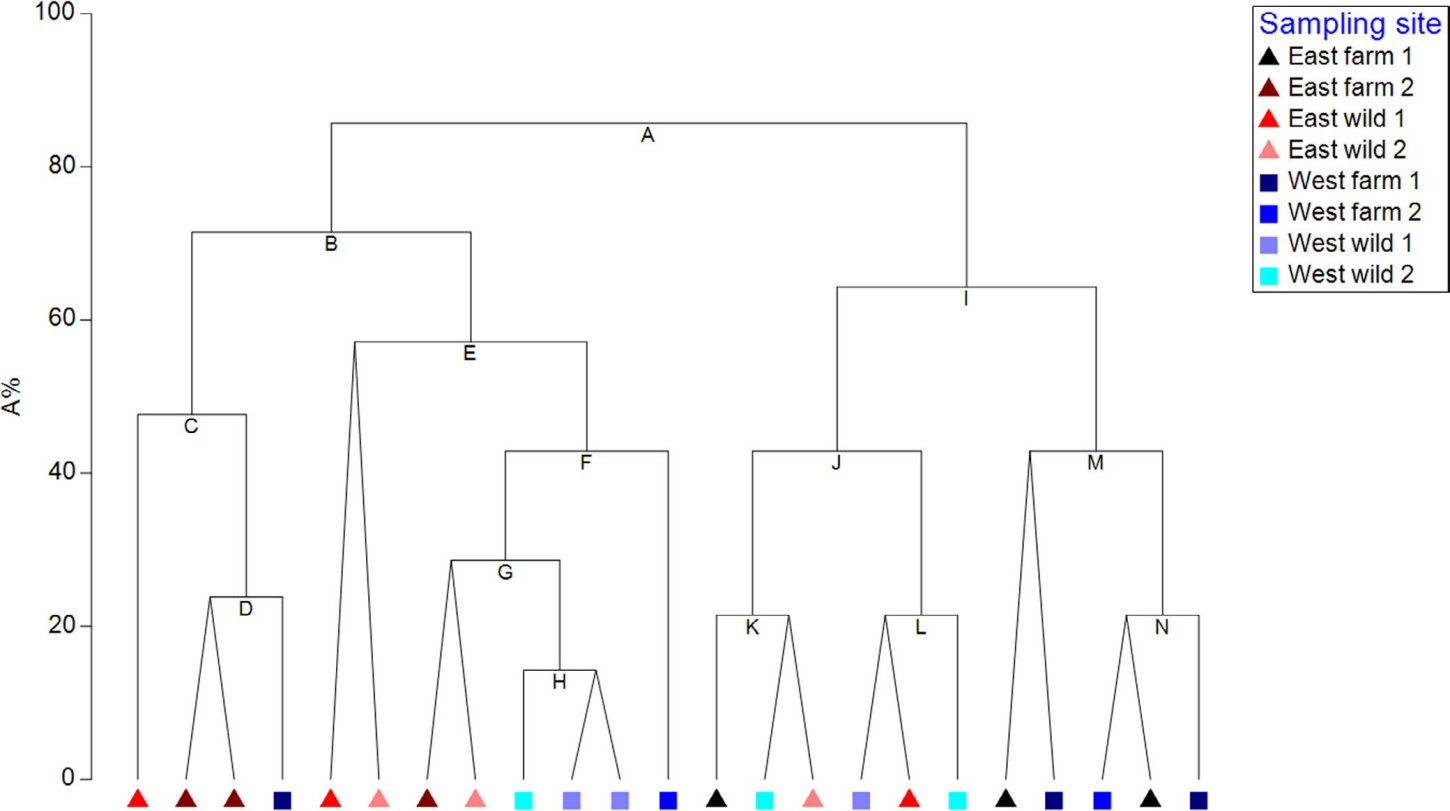
Unconstrained hierarchical divisive UNCTREE clusters based on alpha diversity indices of individual crab gut microbiomes at the genus level. Triangles represent east coast samples and squares represent west coast samples. The dendrogram is plotted against an arbitrary equi‐stepped scale (A%) in which the divisions sum up to 100.

Although microbial composition varied between individuals, all animals from the site EF1 presented consistently high OTU richness and evenness. Yet, in the case of the second farm on the east coast EF2, the OTU richness and evenness were the lowest (Table 2). We clustered samples based on the alpha diversity indices using unconstrained hierarchical divisive clustering routine UNCTREE and obtained two main clusters (Figure 4). The SIMPER analysis showed that the greatest dissimilarity of OTUs present in the eight sites sampled was between farms on the east coast EF1 and EF2 (62.53%) and the farm on the east coast and the wild site on the west coast EF2 and WF2 (64.36%). Examining similarity between wild and farmed animals, it was seen that OTUs varied more in wild animals (66.20% similarity within the group) than in the pond cultivated animals (71.39% similarity within the group).

**TABLE 2:** Alpha diversity indices for individual animals at the genus level.

	Number of taxa	Individuals	Simpson 1‐D	Shannon H	Evenness e^H^/S	Chao−1
EF1C1	215	13299	0.9589	4.05	0.2669	218
EF1C2	245	15040	0.7343	2.521	0.05076	251.3
EF1C3	244	19057	0.879	3.377	0.1201	248.2
EF2C1	92	15504	0.4555	1.109	0.03294	99.5
EF2C2	95	15635	0.452	1.111	0.03198	107.7
EF2C3	125	11575	0.6277	1.923	0.05474	137.7
EW1C1	158	11594	0.7624	2.5	0.07707	182.5
EW1C2	281	14622	0.965	4.228	0.2441	291
EW1C3	57	3144	0.4295	1.05	0.05014	72.4
EW2C1	112	6508	0.6009	1.67	0.04744	149.1
EW2C2	143	12590	0.6397	1.804	0.04249	174.7
EW2C3	252	18948	0.9579	4.072	0.2329	258.1
WF1C1	83	9338	0.4551	1.117	0.03682	99.5
WF1C2	246	12184	0.8933	3.502	0.1349	252.7
WF1C3	262	14370	0.802	2.995	0.07626	263.8
WF2C2	251	15716	0.8471	3.195	0.09721	259.7
WF2C3	185	13056	0.5497	1.587	0.02642	222.6
WW1C1	133	15533	0.5723	1.556	0.03563	177
WW1C2	145	15569	0.6444	1.813	0.04227	178.2
WW1C3	289	13056	0.9627	4.3	0.2549	292.7
WW2C1	141	18257	0.6429	1.801	0.04295	153.2
WW2C2	253	13369	0.9578	4.094	0.2371	263.9
WW2C3	256	19567	0.973	4.337	0.2988	265.8

Simpson's index (1‐D) indicates evenness, Shannon's diversity index (H’) accounts for both species richness and abundance, Buzas and Gibson's evenness index (e^H^/S) implies evenness, Chao1 estimates based on the abundance of less present taxa.

### 3.2 Phenotypic characterization of the gut microbiome

The results of the mapping of obtained OTUs to phenotypic categories showed about 7% of bacteria found in crabs from sites EF1, EW2, and WW2 were potential human pathogens. However, enteric bacteria derived from the gut of warm‐blooded animals and the pathogenic genus like *Salmonella* was less than 0.1% and genera *Staphylococcus* and *Streptococcus* were less than 0.8%. In addition, no crab pathogens such as *Aeromonas*, *Rickettsia*, and *Spiroplasma* were found in any of the samples. Less than 0.1% of OTUs were identified as *Vibrio parahaemolyticus*.

**FIGURE 5:**
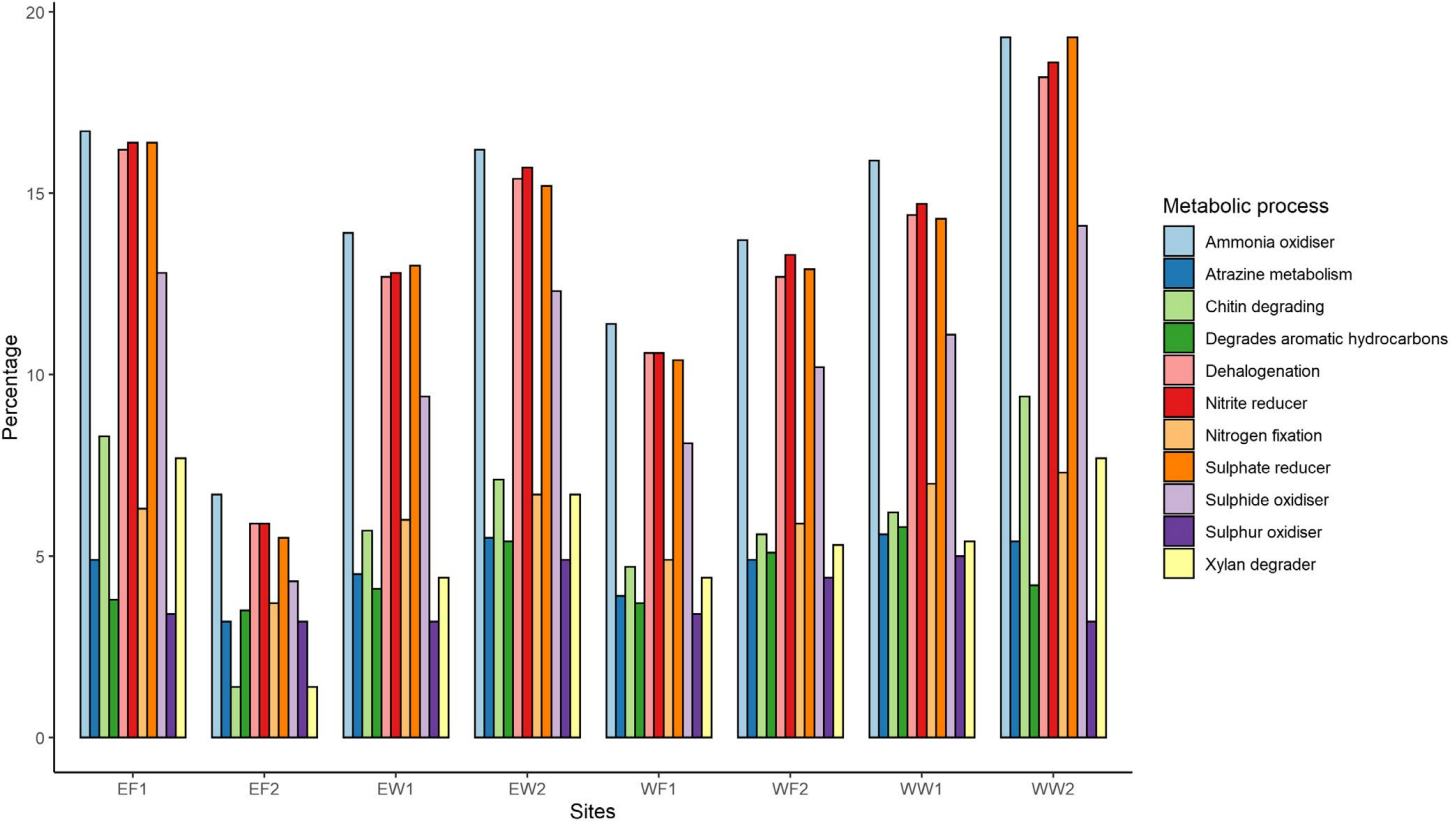
This figure indicates eleven main metabolic processes in which gut bacteria of mud crabs were involved in. Operational taxonomic units OTUs were mapped to phenotypic characteristics with the help of METAGENassist. The results shown, are the average for the sampling site, no individual data were given. To be recognized as one of the eleven main metabolic processes, 5% of OTUs of at least one sample had to be assigned to the process.

Only between 8 to 22% of OTUs on an individual level could be mapped to a specific metabolic pathway. By mapping OTUs to phenotypic characteristics, the main five metabolic processes the mud crab gut microbiome is involved were ammonia oxidation, dehalogenation, sulfate reduction, nitrite reduction, and sulfide oxidation (Figure 5). A very low percentage of lignin degraders were mapped only to wild crab gut samples. Other metabolic processes identified included iron oxidation, lignin degradation, selenate reduction, sulfur reduction, and storage of polyhydroxybutyrate. PERMANOVA showed that temperature (*p* = 0.029) and habitat (*p* = 0.038) significantly affect differences between animals. The coast and salinity did not show any statistically significant difference.

## 4 DISCUSSION

Due to the recognized contribution of the intestinal microbiome to host health, it is essential to assess the bacterial composition of aquaculture species as it plays a significant role in determining their physiological status. Studies on the gut microbiome of aquatic animals and especially fish show that trophic level, season, development, sex, habitat, and life stage are among the factors affecting the composition of the gut microbiome at the interspecies level (Butt & Volkoff, 2019). However, some studies report high individual variability of the crustacean gut microbiome within groups (Ding et al., 2017; Li et al., 2007; Wei et al., 2019). This was observed in this study too and could be explained by the fact that *S. serrata* is an omnivorous and opportunistic scavenger. We did not find any significant differences in the gut microbiome between wild and pond‐cultivated crabs and these results corroborated with the observation in *Eriocheir sinensis* (Li et al., 2007) and black tiger shrimp *Penaeus monodon* (Rungrassamee et al., 2014). On the other hand, higher diversity and higher bacterial load were observed in wild *S. paramamoisam* crabs than in the healthy and diseased pond‐raised crabs (Li et al., 2012). The similarity between groups suggests that environmental conditions might play an essential role in forming the gut microbiome (Fraune & Bosch, 2007). Furthermore, there is no formulated feed for *S. serrata* and the use of probiotics is not common; therefore, wild and pond raised crabs more likely have an identical kind of diet.

The most common phyla in the *S. serrata* gut microbiome were Proteobacteria, Actinobacteria, Cyanobacteria, Firmicutes, and Bacteriodetes, while the studies on *S. paramamoisam* from China found Fusobacteria and Tenericutes to be among the core gut microbiome phyla (Deng et al., 2019; Li et al., 2012; Wei et al., 2019). Yet, in this study, the gut microbiome of *S. serrata* comprised <0.08% Tenericutes, and no Fusobacteria were identified in any of the samples. Fusobacteria, Gram‐negative obligate anaerobic bacilli have been associated with colorectal adenoma and colorectal carcinoma (e.g. Kostic et al., 2012; Saito et al., 2019). Tenericutes, a Gram‐negative obligate cell‐associated bacteria have been recorded in all vertebrate guts examined. Although it is one of the least abundant phyla in mammalian gut microbiota, it has been found in dolphins in a relatively high proportion (Bik et al., 2016). Tenericutes is also one of the most abundant phyla in the gut of the Chinese mitten crab, *Eriocheir sinensis* (Ding et al., 2017; Dong et al., 2018; Zhang et al., 2016). In a meta‐analysis study of marine and freshwater shrimp microbiota, Tenericutes and Fusobacteria were twenty five and five times, respectively, more abundant in marine shrimps compared to freshwater shrimps (Cornejo‐Granados et al., 2018). Estuaries in south India are subject to highly variable salinity due to the heavy monsoon, which can vary from 0 to 35 ppt (Ramachandra et al., 2013; Shruthi et al., 2011), and this could explain the absence of these two phyla in the *S. serrata* gut microbiome. Although variations in the gut microbial composition in different geographical locations are often explained by the differences in the diet and behavior, and not by the location per se (Ye et al., 2014), it is not clear how these differences would affect animal health if crab seed (juveniles for farm rearing) were imported into India, in this instance, from China. Further research is required to determine differences in gut microbial composition at different developmental stages and whether changes in diet and environmental factors induce any alterations. Additionally, it would be interesting to analyze the implications of the above factors on host physiology.

Proteobacteria, Firmicutes, Bacteriodetes, and Actinobacteria comprise core components of the gut microbiome in humans (Hugon et al., 2015; Lawley & Walker, 2013), fish (e.gSandve et al., 2017; Sullam et al., 2012) and crustaceans. However, the crustaceans have less of Actinobacteria (e.g. Ding et al., 2017; Dong et al., 2018; Shi et al., 2019; Zhang et al., 2014, 2016) when compared to the other three groups. The abundance of Cyanobacteria in the gut could be linked with the host trophic level. A study on fish with different diets showed Cyanobacteria to be abundant in filter‐feeding fish, less in herbivorous and omnivorous fish and very little in carnivorous fish (Liu et al., 2016). *Scylla serrata* juveniles and small adult crabs (up to 99 mm CW) are omnivorous, whereas middle‐ and large‐sized crabs are top benthic predators, opportunistic scavengers and exhibit cannibalistic behavior (Alberts‐Hubatsch et al., 2016).

From informal enquiries with crab farmers in India, we are aware that rising temperatures that have been observed in recent years are perceived as one of the reasons for high crab mortality, and ultimately a threat to their livelihoods. Elevated water temperature has been shown to significantly reduce the bacterial diversity in the gut of mussels *Mytilus coruscus*, yet simultaneously increase the abundance of opportunistic bacteria, such as *Bacteroides and Arcobacter*, which could result in higher host susceptibility to disease (Li et al., 2018). Furthermore, the diversity of planktonic bacteria has been found to decrease in the Atlantic Ocean toward the equator (Milici et al., 2016). Thus, as the sea surface temperature (SST) is projected to increase (IPCC, 2014) as a result of global climate change, changes in the crab gut microbiome could be expected, and as a consequence, could negatively affect the physiological and immune status of crabs. This could be detrimental to crabs facing the twin threats of increasing SSTs and increasing pathogen levels such as *Vibrio* spp. due to warm temperatures (Semenza et al., 2017). Yet, the temperature is only one of many environmental factors that could determine microbial richness and abundance, thus more detailed studies considering various physiochemical data are required to understand the role of water temperature in altering the gut microbiome (Thompson et al., 2017). Further investigation is also required to assess the effects of probiotics and other additives such as yeast and jaggery applied in sampling site EF2 in interaction with physiochemical factors.

By mapping OTUs to phenotypic characteristics, almost none of the OTUs were assigned to ammonia‐oxidizing bacteria (AOB) such as *Nitrosphira*, *Nitrosomonas*, and *Nitrosococcus* (Burrel et al., 2001). Thus, we hypothesize that most nitrogen fixation, ammonia oxidation and nitrite reduction in the guts of *S. serrata* is performed by Cyanobacteria as reported in some studies (Andriesse et al., 1990; Herrero et al., 2001) evidence to which is indicated by their significant presence in the gut microbiome. The heterotrophic bacteria, *B. subtilis*, found in soil has also been reported to be involved in nitrogen fixation (Beneduzi et al., 2008; Hashem et al., 2019) and *Bacillus* was one of the main genera found in the crab gut. Ammonia, nitrite, and nitrate are common and essential components in the aquatic environment, yet elevated levels can be toxic to aquatic animals (Romano & Zeng, 2013). Therefore, the results indicate that gut bacteria are strongly involved in mineralization by processing these compounds to avoid toxic effects. Microbial oxidation of sulfur is carried out to produce energy that is further used for synthesizing their structural components and it is possible that *Bacillus* (Friedrich et al., 2001) and *Microcoleus* (Fike et al., 2016) could be responsible for these functions in the crab samples analyzed.

## 5 CONCLUSIONS

This study, to our knowledge, is the first to identify the composition of the gut microbiome of the mud crab, *Scylla serrata* using long read 16S rRNA Oxford Nanopore Sequencing Technology, and assess the impact of geographical location, habitat, and environmental conditions on bacterial diversity and abundance. By comparing the relative abundance and bacterial diversity of crab guts from wild and pond cultivated crabs, from both the east and west coasts of South India, it was observed that the geographical location, habitat, crab body mass and carapace width, and water salinity do not induce changes in the gut microbiome. However, the water temperature was shown to influence gut bacterial diversity, which tended to decrease with increasing water temperature. Human and animal pathogens made up less than 0.1% of the gut samples studied. Thus, the findings suggest that current practices of crab farming result in healthy crabs and that geographical location does not impact farm success. Yet, in the context of climate change, further research is required to assess the effects of temperature on gut microbiomes, and their functions, and whether and how controlling temperature in aquaculture settings might help offset changes associated with variability in climate. In addition to overexploitation, we perceive increased temperature as a result of climate change, to be another potential threat to wild *S. serrata* populations. Furthermore, India has developed a central hatchery for *S. serrata* seed production to promote mud crab aquaculture. The results obtained do not indicate that farmed crabs will be disadvantaged compared to their wild counterparts in terms of their gut microbiome composition.

## CONFLICT OF INTEREST

None declared.

## AUTHOR CONTRIBUTIONS

**Elina Apine:** Conceptualization (supporting); Formal analysis (lead); Investigation (lead); Methodology (equal); Writing‐original draft (lead). **Praveen Rai:** Resources (lead); Supervision (supporting); Writing‐review & editing (equal). **Madhu K Mani:** Investigation (supporting); Project administration (supporting); Resources (equal); Writing‐review & editing (equal). **Vikram Subramanian:** Data curation (lead); Formal analysis (equal); Writing‐review & editing (equal). **Indrani Karunasagar:** Conceptualization (equal); Funding acquisition (supporting); Methodology (equal); Project administration (lead); Supervision (lead); Writing‐review & editing (equal). **Anna Godhe:** Conceptualization (lead); Funding acquisition (supporting); Methodology (lead). **Lucy Turner:** Conceptualization (equal); Funding acquisition (lead); Methodology (equal); Supervision (equal); Writing‐review & editing (lead).

## ETHICS STATEMENT

None required.

## Data Availability

The sequence datasets generated during the current study are available at NCBI Sequence Read Archive (SRA) under BioProject PRJNA691201: https://www.ncbi.nlm.nih.gov/bioproject/PRJNA691201. BioSample accession numbers are SAMN17283444 ‐ SAMN17283464.
